# Diet-Associated Gut Microbiome Divergence Contributes to Phenotypic Differentiation Among Geographically Structured Populations of *Rana dybowskii*

**DOI:** 10.3390/ani16213433

**Published:** 2026-10-30

**Authors:** Linghao Kong, Yi Zhu, Bowen Liu, Hua Wei, Yingdong Li

**Affiliations:** College of Animal Science and Veterinary Medicine, Shenyang Agricultural University, Dongling Road 120, Shenyang 110866, China

**Keywords:** adaptive differentiation, amphibian conservation, chitin degradation, diet metabarcoding, trophic ecology, environmental heterogeneity

## Abstract

Geographic phenotypic variation is a widespread feature of natural populations, often driven by local environmental heterogeneity and trophic interactions. While the gut microbiome is recognized as a key mediator between environmental resources and host physiology, its role in the adaptive differentiation of amphibians remains insufficiently explored. In this study, we employed an integrative approach—combining morphological measurements, DNA metabarcoding for dietary analysis, and 16S rRNA sequencing for gut microbial profiling—to investigate three geographically structured populations (Dandong [DD], Fushun [FS], and Benxi [BX]) of the Dybowski’s frog (*Rana dybowskii*). The results showed that the morphological characteristics (body weight, head-body length, hindlimb length, etc.) of DD were significantly higher than those of FS and BX. Dietary analysis via gut content DNA metabarcoding identified distinct food structures; notably, the DD population consumed a higher proportion of Dipteran prey (e.g., *Smittia*). Correspondingly, the gut microbiota showed significant population-level clustering. We identified an enrichment of specific functional bacteria in the DD population, such as *Arthrobacter*, which is known for its chitin-degrading capabilities, aligning with the consumption of chitin-rich insect prey. Mantel tests further confirmed a strong correlation between dietary composition, microbial community structure, and host morphological traits. These findings suggest that diet-driven gut microbiome variation is consistent with a contributing role in the phenotypic differentiation of *R. dybowskii* across different habitats, although causal relationships remain to be tested directly. By integrating trophic ecology with microbial landscapes, this study provides new insights into the adaptive evolution and resource management of amphibians in heterogeneous environments.

## Simple Summary

Frogs from different geographic locations often differ in body size, but the mechanisms underlying this variation remain unclear. In this study, we examined three populations of Dybowski’s frogs in Liaoning, China, by measuring morphological traits, identifying prey through DNA metabarcoding of gut contents, and profiling gut bacterial communities using 16S rRNA sequencing. We found that frogs from Dandong were larger, consumed more small flies, and harbored a higher abundance of a gut bacterium known to degrade insect exoskeletons. These results link diet, gut microbiota, and host body size and improve our understanding of how amphibians adapt to local environments.

## 1. Introduction

Geographic variation in phenotypes is a widespread feature of natural populations, arising because spatial heterogeneity in climate, resource availability, and habitat structure generates divergent selective pressures that shape intraspecific diversity and drive phenotypic differentiation among populations [1,2]. In amphibians, such geographic variation has been associated with differences in body size, coloration, and life-history traits. For example, the leg coloration of the red-eyed tree frog (*Agalychnis callidryas*) gradually transitions from orange to purple with decreasing latitude [3]. Similarly, studies on Andean poison frogs (*Epipedobates anthonyi*) have shown that environmental differences among populations lead to genetic and phenotypic divergence [4]. In the Japanese tree frog (*Hyla japonica*), geographic barriers such as mountains and open sea contribute to variation in male advertisement call characteristics among populations, independent of habitat or interspecific interactions [5]. Likewise, populations of fire salamander (*Salamandra salamandra*) in northern Iberia exhibit pronounced differences in coloration patterns, body size, and reproductive modes, despite minimal genetic differentiation [6]. These findings highlight the key role of environmental heterogeneity in driving morphological divergence among amphibian populations.

Diet represents a critical ecological interface linking environmental heterogeneity to host physiology and phenotype [7]. Habitat-specific prey availability can influence not only nutritional intake but also the composition and function of the gut microbiome, which mediates nutrient assimilation and metabolism [8,9]. Studies in amphibians have demonstrated that variation in diet can shape gut microbial communities, which in turn affect host metabolism, immunity, and adaptation [10]. As such, diet–microbiome interactions provide a plausible mechanistic pathway through which geographic differences in resource availability may be translated into phenotypic divergence.

Despite growing interest in the ecological roles of host-associated microbiomes, the contribution of diet-driven gut microbiome variation to phenotypic differentiation among geographically structured amphibian populations remains poorly understood. Most existing studies have been largely descriptive or restricted to single populations, often focusing on gut microbial composition or host traits independently, without explicitly linking diet, microbiome structure, and phenotypic variation across spatial scales. Geographic variation can influence the availability and composition of prey communities, which in turn can shape host diet and the associated gut microbiome, providing a potential pathway through which environmental heterogeneity drives phenotypic divergence. For example, the diet of the aposematic toad (*Melanophryniscus rubriventris*) in northwestern Argentina comprises 17 arthropod categories, including ants, mites, aphids, burrowing bugs, and beetles, with the relative contribution of each prey type varying among populations [11]. Such interpopulation dietary variation illustrates how local prey availability can translate into differences in nutrient intake and associated physiological or chemical traits. However, integrative studies linking diet, gut microbiota, and phenotypic variation in free-ranging amphibian populations are still emerging, because the combined application of prey DNA metabarcoding, microbial 16S rRNA profiling, and morphometric analysis has only recently become technically feasible; accordingly, the extent to which population-level differences in prey consumption correspond to shifts in microbial communities and, ultimately, to morphological divergence remains unclear. Understanding these relationships is critical for elucidating the mechanisms by which environmental heterogeneity and trophic interactions mediate intraspecific variation and local adaptation in amphibians.

*Rana dybowskii* is an amphibian endemic to Northeast China, the Russian Far East, and the Korean Peninsula, where it primarily inhabits the mountainous regions of the Changbai Mountain range and its foothills. A previous phylogeographic study of the *R. dybowskii* species complex [12] revealed deep lineage divergence associated with the orogenic history of this region, although population-scale genomic data across its range are still limited. This species holds both significant ecological and economic value, and recent expansion of its farming industry has raised concerns about reduced genetic diversity due to translocations and introductions [13]. In addition, environmental factors such as shelter, hibernation, and light spectrum have been shown to affect behavioral rhythms, physiology, and gut microbiota in this species [14,15,16]. Preliminary observations in *R. dybowskii* indicate pronounced differences in body size and limb morphology among geographically separated populations, suggesting that local environmental conditions may influence growth and development [17]. Despite its importance, such integrative studies are still lacking for this species, as the combined tools for diet, microbiota, and phenotypic analysis have only recently become available. In this study, we aimed to evaluate the differences in diet and intestinal microbial communities among three geographically distinct populations of *R. dybowskii* and to test whether diet-driven variation in gut microbiota underlies observed phenotypic differentiation. Using 16S rRNA gene sequencing and DNA metabarcoding of gut contents, we characterized microbial composition and dietary profiles across populations, linking these factors to growth- and size-related traits. By revealing how diet-mediated microbial variation contributes to phenotypic divergence, this work provides critical insights into the mechanisms by which environmental heterogeneity shapes intraspecific diversity and offers practical guidance for conserving local germplasm and optimizing breeding programs for this species.

## 2. Materials and Methods

### 2.1. Ethics Statement

Although *Rana dybowskii* is listed as “Near Threatened” on the IUCN Red List, in China, it is regarded as an important economic frog species and is neither endangered nor nationally protected. All experiments were performed in accordance with the guidelines for scientific purposes, animal care, and use formulated by the Animal Ethics Committee of Shenyang Agricultural University (protocol code No. 202403081 and date of approval 20 October 2024). All animal surveys were conducted in compliance with the guidelines established by the Shenyang Agricultural University Experimental Animal Management Committee (SYAEAMC).

### 2.2. Sample Collection and Morphological Measurement

Adult *R. dybowskii* were collected in April 2025 from three geographical populations in Liaoning Province, China—Dandong (DD), Fushun (FS), and Benxi (BX)—with environmental parameters for 2023–2025 obtained from the local Bureau of Statistics to characterize habitat heterogeneity among the sampling sites (Table 1, Figure S1). A total of 1254 adult individuals (362 from BX, 478 from DD, and 414 from FS) were transported on ice to the Animal Breeding Laboratory of Shenyang Agricultural University for morphological measurements. To minimize measurement error and ensure consistency among populations, all morphological measurements were performed in the laboratory by a single observer using the same instruments, including body weight, snout-vent length, head length, head width, snout length, forearm and hand length, forearm width, hindlimb length, tibia length, foot length, and thigh length. After measurement, 16 adult frogs (8 females and 8 males) were randomly selected for gut microbiota and dietary analysis by 16S rRNA amplicon sequencing and DNA metabarcoding, respectively. Specifically, 6 frogs (3 composite samples, each with one female and one male) were used for gut microbiota analysis, and the remaining 10 (5 composite samples) for dietary analysis. The remaining 1206 individuals were euthanized and dissected, and liver, gastrointestinal tract, and muscle samples were stored at −80 °C for DNA extraction and other tissue analyses. All individuals were euthanized by rapid cooling in an ice-water bath (hypothermia) until loss of the righting reflex, followed by double pithing (destruction of the brain and spinal cord) to ensure death, in accordance with the protocol approved by the SYAEAMC.

### 2.3. Extraction of Intestinal Microbial DNA and 16S rRNA Sequencing

Genomic DNA was extracted from the intestinal contents using the OMEGA Soil DNA Kit (M5635-02, OMEGA Bio-Tek, Norcross, GA, USA) following the manufacturer’s instructions. For gut microbiota characterization, the V3-V4 region of the bacterial 16S rRNA gene was amplified using primers 338F (5′-ACTCCTACGGGAGGCAGCA-3′) and 806R (5′-GGACTACHVGGGTWTCTAAT-3′), following the standard Illumina amplicon protocol [18]. PCR products were checked on a 1% agarose gel and sequenced using paired-end Illumina NovaSeq PE250 technology (Personal Biotechnology Co, Ltd., Shanghai, China.). Sequence processing and ASV inference were performed with the QIIME2 2024.10 platform and the DADA2 plugin [19], including demultiplexing (q2-demux), primer trimming (q2-cutadapt), denoising, merging, and chimera removal. Taxonomic assignment of ASVs was performed against the SILVA 138 reference database. Alpha diversity indices (Chao1, observed ASVs, Shannon, Simpson) and beta diversity (Bray–Curtis dissimilarity, visualized by PCoA and NMDS) were calculated in QIIME2 and R v4.3.1.

### 2.4. Analysis of Intestinal Microbial Alpha/Beta Diversity

Bioinformatics analysis of the microbiome was performed using QIIME2 version 2024.10 [19], strictly following the protocols outlined in the official guidelines (available at https://docs.qiime2.org/2024.10/tutorials/overview/, accessed on 4 January 2026) with minor adjustments. Initial processing of raw sequencing data included demultiplexing using the q2-demux plugin, followed by primer removal using the q2-cutadapt plugin [20]. Sequencing reads were processed through filtering, denoising, merging, and chimera removal, all accomplished by applying the DADA2 plugin [21]. Taxonomic assignment of ASVs was performed against the SILVA database [22] using the q2-feature-classifier plugin [23]. Sequence data analysis was conducted using QIIME2 and R v4.3.1. Alpha diversity (including Chao1, observed species, Shannon, and Simpson indices) was calculated at the ASV level using the ASV table from QIIME2 and presented via box plots. ASV abundance and diversity among samples were compared using rarefaction curves. For beta diversity, which was used to study differences in microbial communities between samples, the Bray–Curtis dissimilarity index was employed, and the results were visualized through Principal Coordinates Analysis (PCoA), Non-metric Multidimensional Scaling (NMDS), and hierarchical clustering. Venn diagrams were generated using the “Venn Diagram” R package to show shared and unique ASVs among samples or groups based on the presence of ASVs in different samples and groups (regardless of their relative abundance).

### 2.5. Analysis of Dietary Composition

For dietary analysis, the entire gastrointestinal tract (stomach + intestine) was sampled and pooled, as undigested prey in the stomach completes the recent-feeding record. Intestinal contents from the selected frogs were used to characterize diet composition via gut content DNA metabarcoding [24]. Genomic DNA was extracted as described above. The mitochondrial COI gene was amplified using a modified version of the arthropod-targeting primer pair NoPlantF_270 (5′-RGCHTTYCCHCGWATAAAYAAYATAAG-3′)and mICOIintR_W (5′-S-GRGGRTAWACWGTTCAWCCWGTNCC-3′), which was designed to enrich arthropod DNA while suppressing plant DNA amplification [25], targeting a ~250–300 bp fragment suitable for insect identification. PCR amplifications were performed in 25 μL reactions under standard cycling conditions (initial denaturation at 94 °C for 3 min; 35 cycles of 94 °C for 30 s, 50 °C for 45 s, 72 °C for 45 s; final extension at 72 °C for 10 min).

PCR products were purified and pooled equimolarly for library preparation, then sequenced on an Illumina PE150 platform (paired-end 150 bp reads) (Lingen Biotechnology Co, Ltd., Shanghai, China). Raw sequencing reads were quality-filtered, merged, and denoised using QIIME2 v2024.10 with the DADA2 plugin to generate Amplicon Sequence Variants (ASVs). For taxonomic assignment, ASVs were compared against a reference COI database using BLAST (v2.12.0+), with sequence identity ≥ 95% and coverage ≥ 90% required for reliable identification. ASVs with 100% identity to a single local species were assigned to species level; sequences matching multiple species or non-local taxa were assigned to the lowest shared taxonomic rank (genus or family). Sequences with <95% identity were classified as “unknown.”

For downstream analyses, ASVs representing the same taxonomic entity were grouped into Molecular Operational Taxonomic Units (MOTUs), following established methods [24]. Only ASVs/MOTUs assigned to Arthropoda (Insecta) were retained for the downstream dietary analyses; ASVs assigned to non-arthropod taxa were excluded. DNA metabarcoding of gut contents has been successfully applied to characterize amphibian diets, including species-level prey identification [26]. This approach allowed a standardized assessment of prey composition across populations, highlighting interpopulation variation in insect prey diversity and relative abundance.

### 2.6. Correlation Analysis Between Diet, Microbiota, and Host Phenotype

To explore the relationships among diet composition, gut microbial communities, and host phenotypic traits, we performed multivariate statistical analyses. Relative abundances of dietary MOTUs and microbial ASVs were first Hellinger-transformed to reduce the influence of highly abundant taxa. Redundancy analysis (RDA) and canonical correspondence analysis (CCA) were conducted using the R package vegan v2.6-4 to evaluate the effects of diet on microbial community structure. Spearman’s rank correlation coefficients were calculated to assess associations between the relative abundances of dominant prey taxa and key bacterial taxa, as well as between microbial composition and morphological traits (e.g., body weight, head-body length, hindlimb length). Significance of correlations was determined using false discovery rate (FDR) correction for multiple testing. In addition, Mantel tests were used to evaluate congruence between pairwise distance matrices of diet, microbiota, and host morphological traits across populations.

### 2.7. Data Processing and Statistical Analysis

All statistical analyses were performed in R v4.3.1 [27] and SPSS v27.0 [28]. Morphological data were tested for normality and homogeneity of variance prior to analysis. One-way ANOVA followed by Duncan’s multiple comparison test was used to compare morphological traits among populations and sexes. Microbial alpha diversity indices (Chao1, Shannon, Simpson) and dietary richness were compared using Kruskal–Wallis tests when normality assumptions were not met. Beta diversity analyses, including Bray–Curtis dissimilarity, were visualized using PCoA and NMDS. Heatmaps, Venn diagrams, and network visualizations were generated in R using the packages ggplot2 [29], pheatmap [30], igraph [31], vegan [32], and VennDiagram [33], respectively, to depict patterns of microbial and dietary variation. Results are presented as mean ± standard deviation (SD), and differences were considered statistically significant at *p* < 0.05.

## 3. Results

### 3.1. Gut Microbial Community Composition

**Figure 1:**
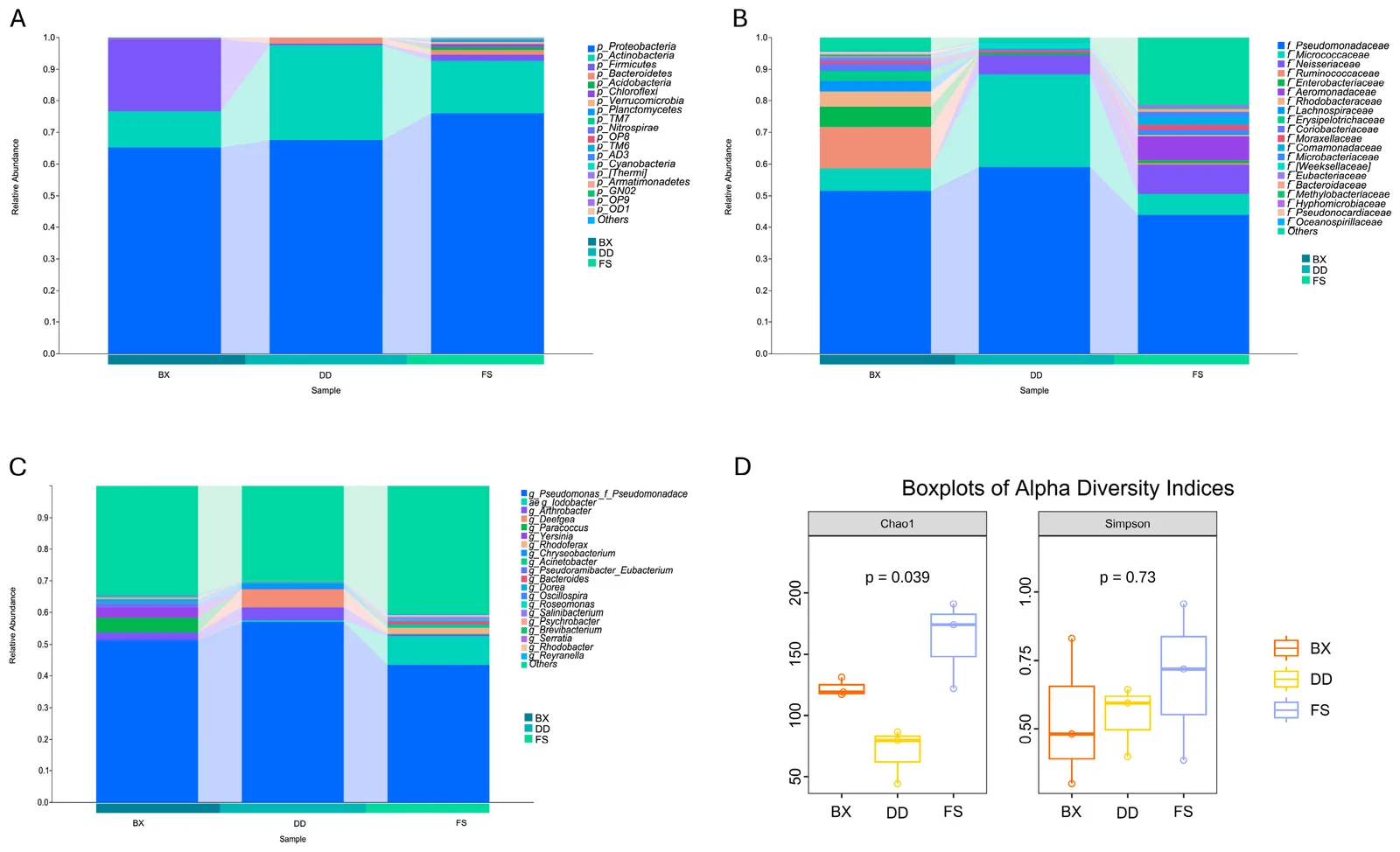
Relative abundance of intestinal microbial communities of *Rana dybowskii* from different geographical populations. (**A**) Phylum level. (**B**) Family level. (**C**) Genus level. (**D**) Alpha diversity index.

At the phylum level, the gut microbiota across all samples was dominated by four major bacterial phyla (relative abundance > 1%), Proteobacteria, Actinobacteria, Firmicutes, and Bacteroidetes, collectively accounting for over 98.6% of the total microbial community (Figure 1A). At the family level, Pseudomonadaceae emerged as a key dominant family, and Micrococcaceae in the DD group was higher than in other groups (Figure 1B). This compositional shift was further reflected at the genus level, where *Pseudomonas* was identified as the overall dominant taxon, complemented by the enrichment of *Iodobacter* and *Arthrobacter* in the DD group (Figure 1C). Regarding alpha diversity, while the Simpson index remained stable across groups (*p* = 0.73), the Chao1 index revealed significant geographic variation (*p* = 0.039), with the highest microbial richness in the FS population, intermediate in BX, and the lowest in DD (Figure 1D). Furthermore, Principal Coordinates Analysis (PCoA) based on Bray–Curtis distances demonstrated distinct clustering of the microbial communities (PC1: 30.1%, PC2: 21.8%), confirming that geographic location is a significant driver of gut microbial beta diversity in these forest frogs (Figure S2).

### 3.2. Dietary Composition

**Figure 2:**
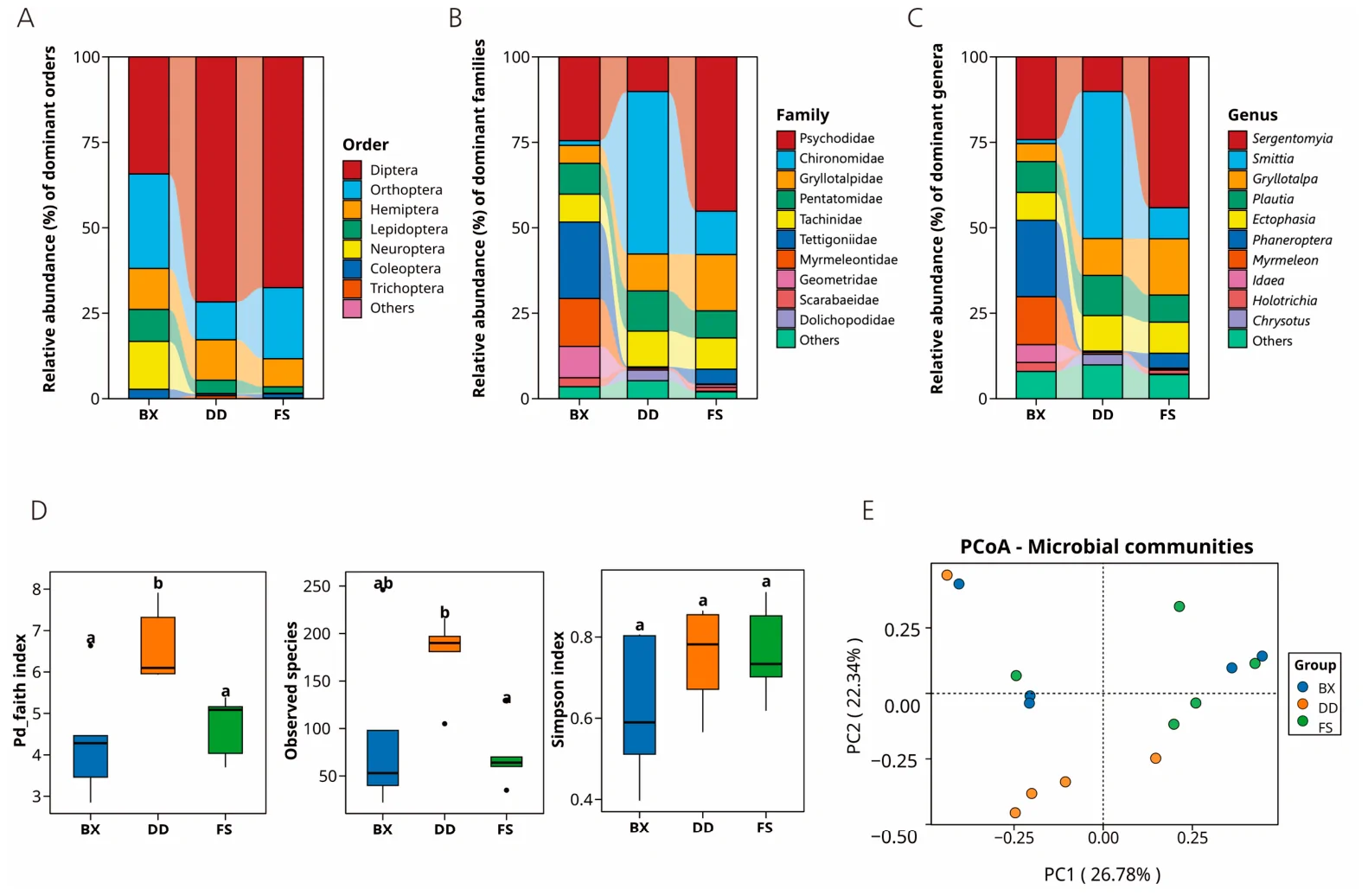
Insect composition of the intestine of *Rana dybowskii* from different geographical populations. (**A**) Order level. (**B**) Family level. (**C**) Genus level. (**D**) Alpha diversity index. (**E**) Beta diversity index.

At the order level, Diptera accounted for the highest proportion in all three populations, whereas Neuroptera showed the highest proportion in BX and lower proportions in DD and FS (Figure 2A). At the family level, *Psychodidae* was the most abundant family in both the FS and BX populations, whereas *Chironomidae* was dominant in the DD population (Figure 2B). At the genus level, *Sergentomyia* had the highest relative abundance in FS, while *Smittia* and *Chrysotus* were dominant in DD but nearly absent in BX and FS (Figure 2C). In BX, *Phaneroptera* and *Sergentomyia* jointly contributed to a large proportion of the diet. At the species level, *Smittia aterrima* exhibited a disproportionately high relative abundance in the DD population compared to the FS and BX groups (Figure S3). In alpha diversity analyses, the DD group exhibited significantly higher values than both the FS and BX groups in the Pd_faith index (*p* < 0.05). Also, in the Observed species, DD was significantly higher than FS. No significant differences were detected among populations in the Simpson index (Figure 2D). In beta diversity, PCoA of dietary compositions among the populations showed (PC1: 26.78%, PC2: 22.34%), a pattern consistent with that observed for their microbial communities (Figure 2E).

**Figure 3:**
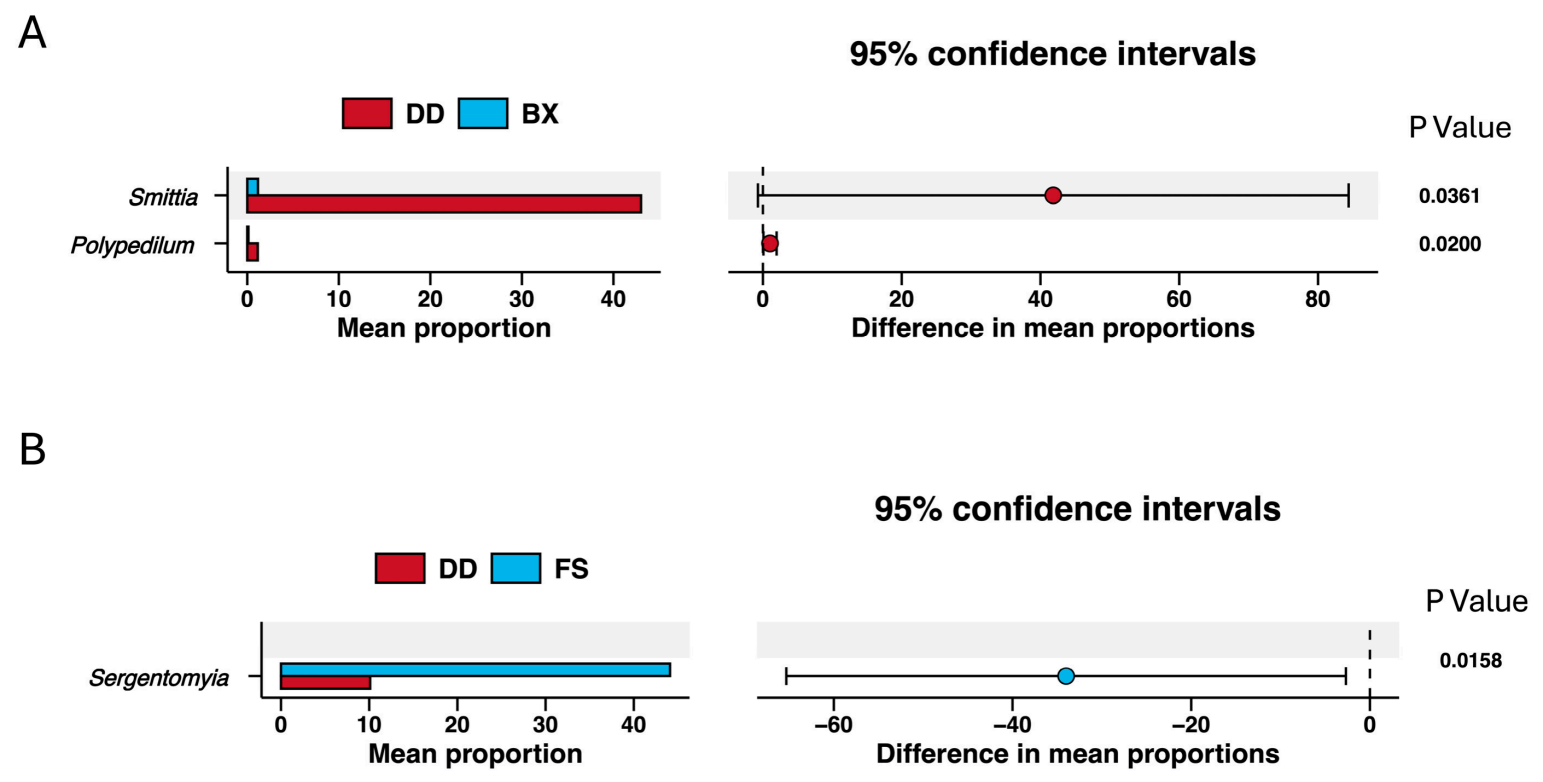
Wilcoxon rank-sum test of insect composition of *Rana dybowskii* from different geographical populations (**A**) DD vs. BX. (**B**) DD vs. FS.

According to the Wilcoxon rank-sum test, the DD population was characterized by a significant enrichment of chironomid prey at the genus level, particularly *Smittia* and *Polypedilum*, compared to the BX population (Figure 3A). *Polypedilum* occurred at very low relative abundance (<1%) in all populations and was therefore not displayed among the dominant genera in Figure 2C, although it was significantly enriched in the DD population in the differential abundance analysis (Figure 3A). In contrast, the FS population exhibited a significantly higher relative abundance of *Sergentomyia* than the DD group (Figure 3B).

### 3.3. Morphological Differentiation and Environmental Contexts

Morphological comparisons revealed significant phenotypic divergence among the three *R. dybowskii* populations (Table 2). In females, the DD population consistently displayed the largest body size, with significantly higher values in body weight, snout-vent length (SVL), head length, snout length, forearm and hand length, forearm width, foot length, and thigh length compared to both FS and BX populations (*p* < 0.05). For hindlimb length and shank length, DD and BX females were statistically comparable and both significantly exceeded FS females. In males, the DD population also showed greater morphological trait values. DD males exhibited significantly greater body weight, SVL, head width, snout length, forearm and hand length, hindlimb length, shank length, and thigh length than their BX counterparts (*p* < 0.05), whereas forearm width was comparable between DD and BX. Compared with FS males, DD males had significantly greater SVL, head width, forearm and hand length, forearm width, and shank length (*p* < 0.05).

This more pronounced phenotypic differentiation in the DD population coincided with distinct regional environmental profiles (Table 1). Environmentally, DD exhibited the highest average annual temperature (10.1 °C) and the highest rainfall (1322.1 mm) and relatively high forest coverage (74.5%), whereas FS had the lowest rainfall (786.7 mm) and temperature (7.2 °C) but the longest sunshine duration (2367.2 h). The combination of a warmer climate and moderate resource availability in the DD habitat may contribute to the enhanced body condition observed in this population, potentially linking environmental factors to the previously documented differences in gut microbiota and dietary composition.

### 3.4. Determinants of Gut Microbial and Dietary Composition Structures

**Figure 4:**
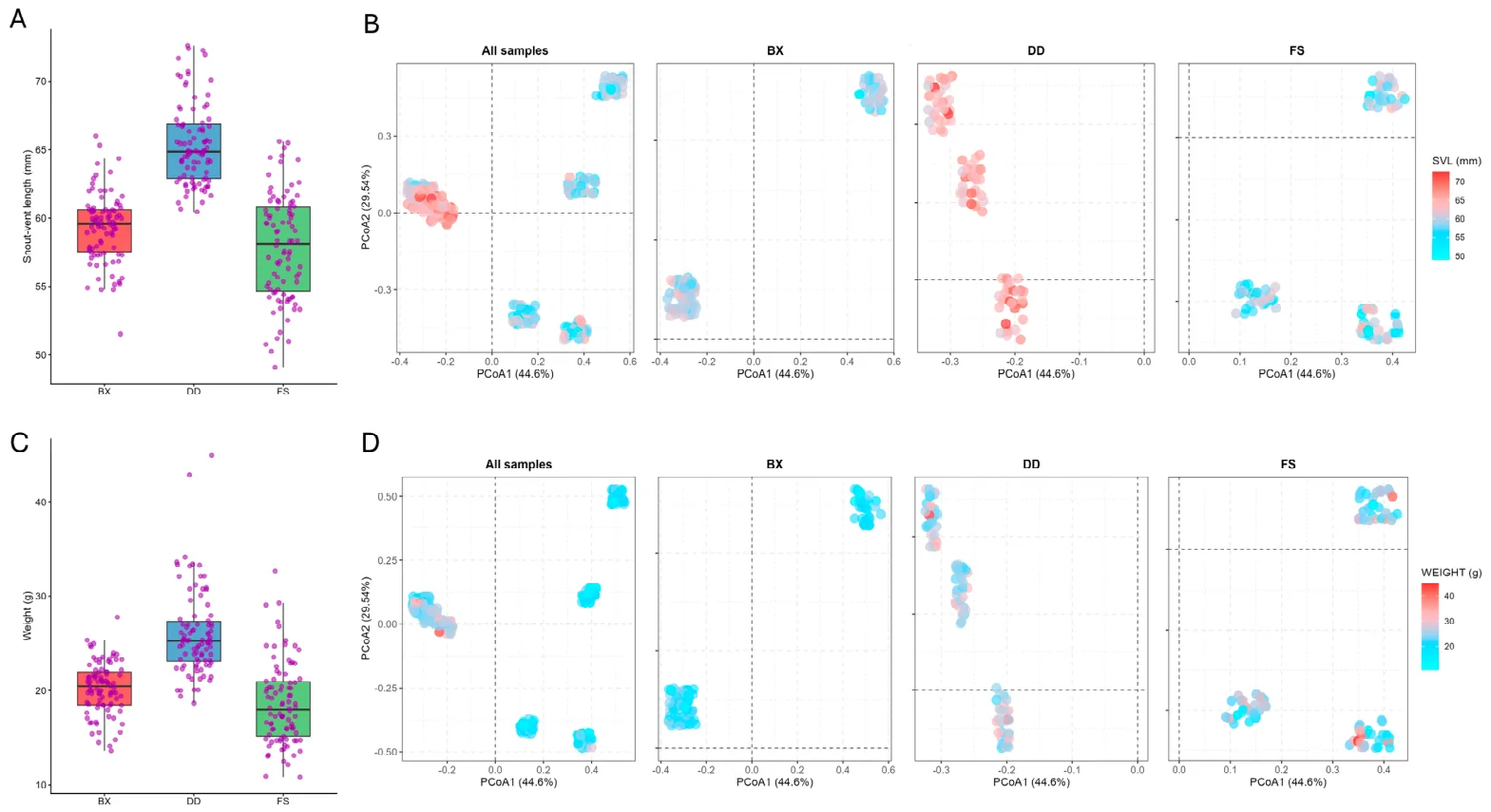
Influence of geography on gut microbial composition. (**A**) Snout−vent length (SVL) of each frog population. (**B**) PCoA scatterplots showing the SVL−dependent distribution of samples across locations, and within DD, FS and BX. (**C**) Body weight of each frog. (**D**) PCoA scatterplots showing the body weight -dependent distribution of samples across locations, and within DD, FS and BX.

To quantify the relative contributions of host and environmental factors to biological divergence, PERMANOVA based on Bray–Curtis distances was conducted. A strong population effect was detected for both gut microbiota (R^2^ = 0.4237, *p* = 0.001; Table S1) and dietary composition (R^2^ = 0.4635, *p* = 0.001; Table S2), indicating that geographic grouping accounted for a substantial proportion of variation. As shown in Figure 4A,C, the DD population exhibited significantly higher SVL and Body Weight (*p* < 0.05) with tightly clustered distributions, whereas the BX population showed markedly greater dispersion in both traits (Figure 4B,D). The FS population displayed intermediate patterns.

**Figure 5:**
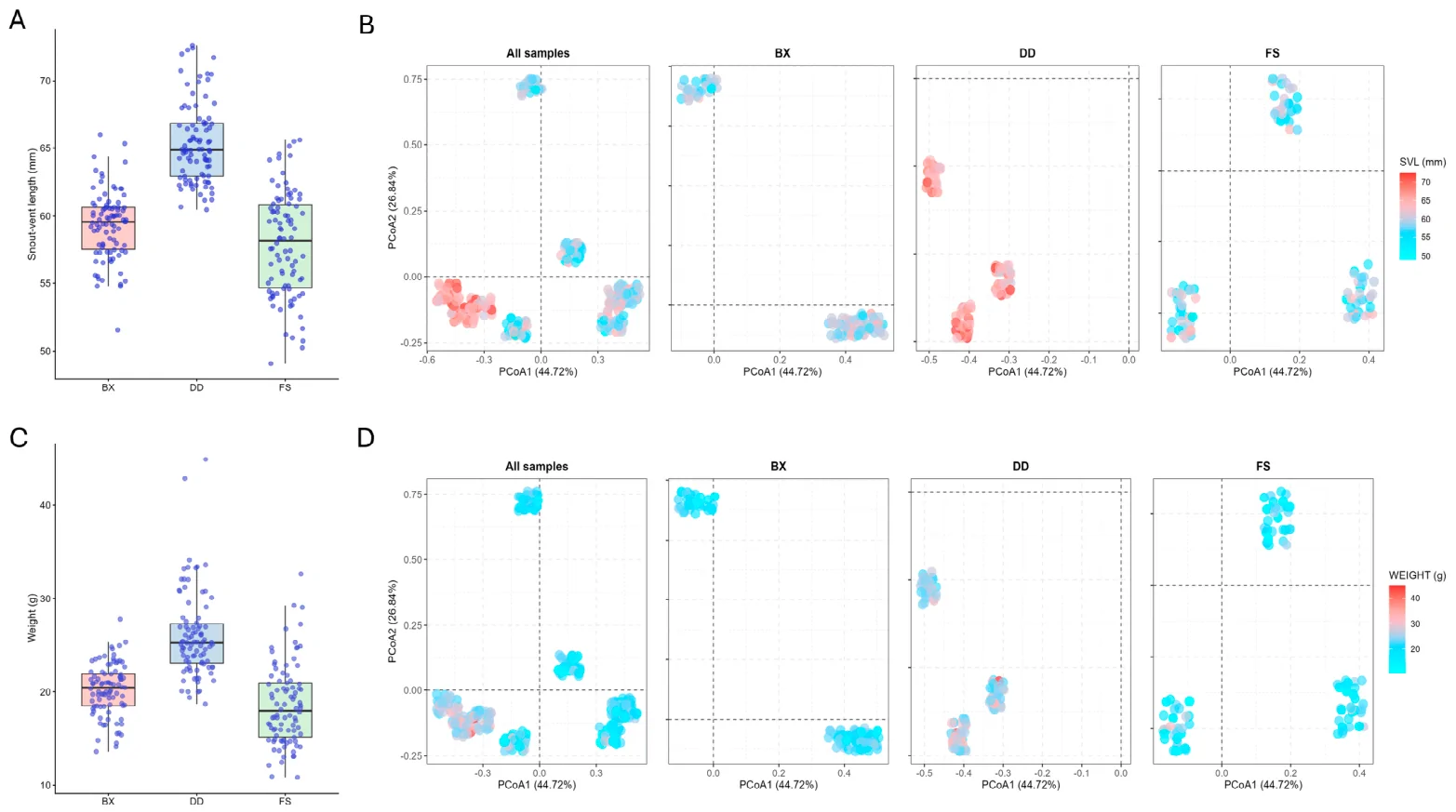
Influence of geography on dietary arthropod composition. (**A**) Snout−vent length (SVL) of each frog population. (**B**) PCoA scatterplots showing the SVL−dependent distribution of samples across locations, and within DD, FS and BX. (**C**) Body weight of each frog. (**D**) PCoA scatterplots showing the body weight-dependent distribution of samples across locations, and within DD, FS and BX.

Dietary composition also differed among populations. The DD group formed a compact cluster in ordination space (Figure 5A,C), while the BX group occupied a broader multivariate space with pronounced scatter (Figure 5B,D), and FS showed partial overlap with both groups. Within-population analyses revealed that Body Weight significantly explained variation in both gut microbiota and dietary composition only in the BX population, whereas SVL showed no significant effects (Tables S1 and S2). No significant associations were detected in the DD or FS populations. These results indicate that population differentiation is the primary driver of microbial and dietary variation, with host-related effects detectable only under conditions of increased phenotypic variability.

### 3.5. Coupling Between Dietary Composition and Gut Microbiota

**Figure 6:**
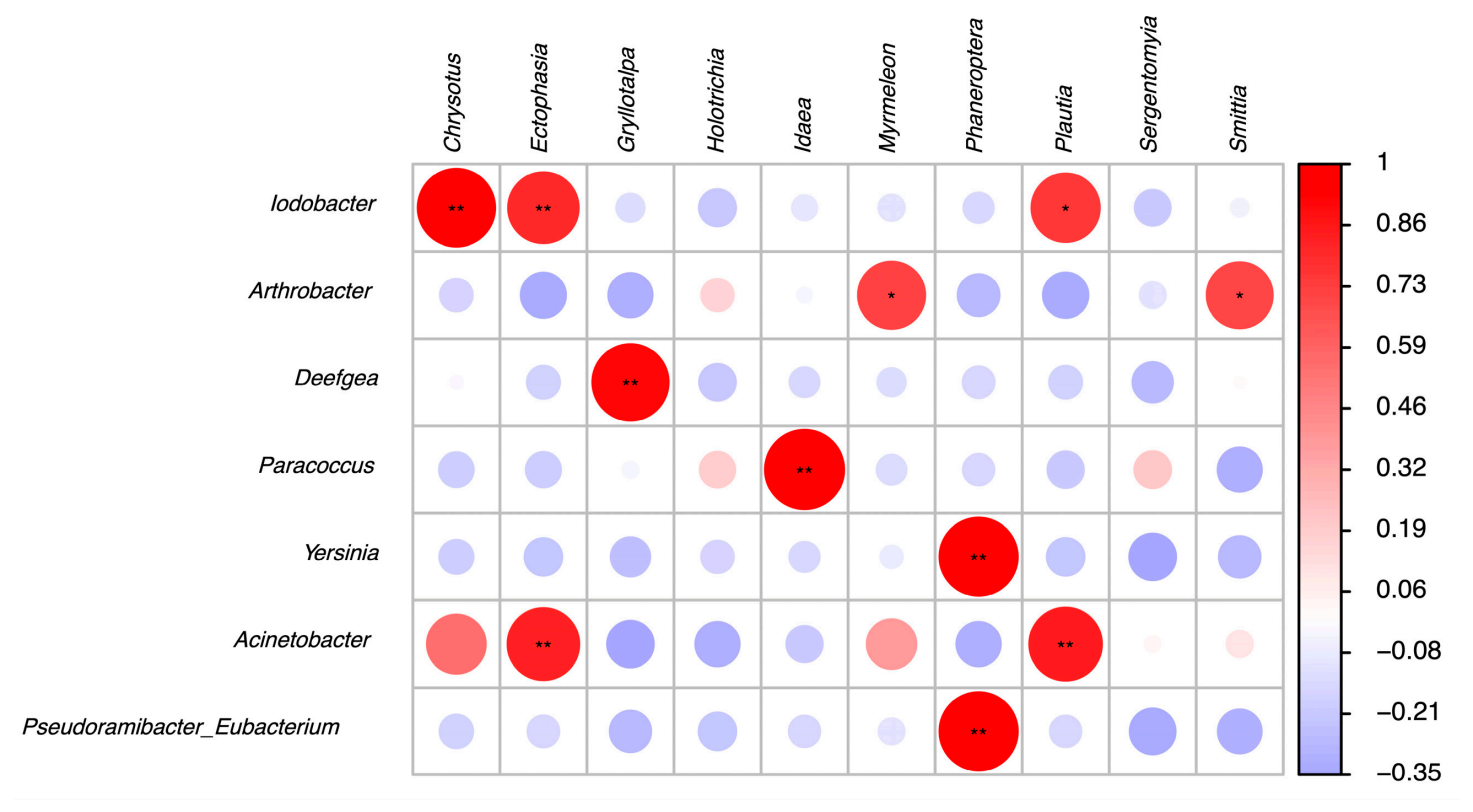
Correlations of top 10 dietary arthropod and gut microbial in genera level. *: *p* < 0.05; **: *p* < 0.01.

To further explore the linkage between dietary composition and gut microbial communities, correlation analyses were performed between dominant dietary taxa and bacterial genera (Figure 6) and species (Figure S4). Several bacterial taxa, including *Iodobacter*, *Arthrobacter*, and *Deefgea*, exhibited strong correlations with specific dietary insects, particularly chironomid-related taxa such as *Smittia* spp. and *Polypedilum* spp., which were highly enriched in the DD population. In contrast, dietary taxa characteristic of the FS and BX populations, such as *Sergentomyia* and *Gryllotalpa*, as well as *Myrmeleon* and *Phaneroptera*, showed fewer and more variable associations with gut microbial taxa, involving genera such as *Paracoccus*, *Acinetobacter*, and *Yersinia*. Notably, both FS and BX populations exhibited relatively weak and inconsistent diet–microbiota associations compared to the DD population, indicating a reduced strength of diet-driven microbial structuring.

## 4. Discussion

### 4.1. Ecological Significance of Phenotypic Differentiation in Geographical Populations

Geographic and environmental factors drive phenotypic differentiation among populations of amphibians. In northern Madagascar, three populations of the frog *Mantidactylus bellyi* generally show significant morphological differences, and the differences in head-body length are consistent with the results of genetic subpopulations [34]. Studies such as [17] have shown that the morphological characteristics of *Rana dybowskii* have significant geographical variations. In this study, morphological traits showed consistent trends between males and females across the three regions, with individuals from Dandong exhibiting significantly larger body size and limb measurements compared to those from Fushun and Benxi, which were relatively similar overall despite differences in specific traits such as hindlimb length and forearm width. This study provides an integrative assessment linking environmental variation with phenotypic traits across geographically structured populations of *R. dybowskii*, highlighting the potential role of habitat conditions in shaping morphological divergence.

Although all three regions are located along the southwestern extension of the Changbai Mountain Range and share a temperate monsoon climate, environmental conditions differ in ways that may influence growth and development. In this study, the larger body size and more pronounced phenotypic differences of the Dandong population corresponded closely with its distinct environmental profile, characterized by the highest mean annual temperature (10.1 °C), the highest precipitation (1322.1 mm), and substantial forest coverage (74.5%). In contrast, Fushun exhibited lower temperature and precipitation but longer sunshine duration, while Benxi showed the highest forest coverage (78.94%) among the three sites and higher precipitation than FS. Such environmental heterogeneity may influence growth through multiple pathways. Warmer temperatures can accelerate metabolic processes and growth rates, while higher rainfall and moderate moisture conditions support higher availability of insect prey resources [35]. Previous studies have demonstrated that larval growth rate and post-metamorphic body size are strongly shaped by food availability and ecological pressures such as predation and competition [36,37]. Notably, the warm and humid conditions in Dandong, combined with the abundant chironomid prey in its aquatic habitats, may contribute to the enhanced body size observed in this population.

### 4.2. Diet as a Key Factor Driving the Differentiation of Gut Microbiota

The observed differences in dietary composition among the DD, FS, and BX populations reflect pronounced geographic variation in prey availability and foraging strategies. The DD population is characterized by the highest annual precipitation and mean temperature, which may promote the development and abundance of aquatic and semi-aquatic insects such as *Smittia*, *Polypedilum* and *Chrysotus*, given their dependence on relatively warm and stable aquatic environments for larval development [38,39]. Meanwhile, the higher forest coverage in BX likely favors the proliferation of *Phaneroptera*, as this genus is known to prefer forest edges, shrublands, and wooded habitats [40]. Meanwhile, the FS population, characterized by lower precipitation and forest coverage but higher solar radiation, represents a drier and more open habitat. These conditions are conducive to soil- and vegetation-associated insects, such as *Sergentomyia* and *Gryllotalpa*, resulting in a dietary shift toward terrestrial prey [41,42]. Collectively, these findings suggest that temperature and moisture availability jointly shape prey community structure, leading to a gradient in dietary composition from aquatic-dominated systems (DD) to terrestrial-dominated systems (FS and BX), with BX exhibiting an intermediate but structurally distinct pattern.

This aquatic-to-terrestrial dietary gradient suggests that *R. dybowskii* exhibits considerable trophic flexibility, with diet composition varying according to local resource availability and seasonal environmental conditions [43]. In DD habitats, the seasonal emergence of aquatic chironomids may represent a biomass-rich resource pulse, whereas terrestrial prey in FS and BX may provide more stable but less concentrated energy inputs [44]. Because arthropod prey contains abundant chitinous materials, differences in prey composition may further influence gut microbial functions associated with chitin degradation. Therefore, variation in local climate, prey availability, and gut microbial composition may jointly contribute to population-level differences in growth and body size [45]. Moreover, there is a strong positive correlation between *Arthrobacter* and *Smittia* in the DD population. The genus *Arthrobacter* is well known for its chitinolytic capabilities, producing extracellular enzymes such as chitinases and chitin deacetylases that enable the efficient degradation of crystalline chitin into utilizable carbon and nitrogen sources [46,47]. Although *Smittia* exhibits relatively low chitin content per individual, chironomid taxa are characterized by rapid population growth and synchronous mass emergence, generating substantial prey biomass over short time periods [48,49]. This high intake-driven chitin flux, combined with the relatively soft exoskeleton of *Smittia* that enhances substrate bioavailability, may collectively promote the enrichment of chitin-degrading bacteria such as *Arthrobacter* in the gut microbiota. This pattern suggests that transient but high-magnitude resource pulses can restructure microbial communities of *R. dybowskii*. This process may enhance the frog’s capacity to utilize temporally variable food resources, thereby contributing to ecological adaptation across heterogeneous environments. Consistently, the DD population exhibited the highest snout–vent length (SVL) and body weight, implying that enhanced utilization of pulsed dietary resources may translate into improved growth performance.

In the BX population, the relative abundance of *Yersinia* in the gut microbiota, as well as that of its associated prey taxon *Phaneroptera*, was higher than in the DD and FS populations, and a significant positive correlation was observed between them. Comparative genomic studies have shown that *Yersinia* shares a suite of insect-associated virulence and adaptation genes with the entomopathogenic bacterium *Photorhabdus*, including unique two-component regulatory systems, quorum-sensing pathways, and metabolic traits that facilitate proliferation within insect hemolymph [50]. The frequent consumption of *Phaneroptera* by frogs in the BX region may therefore promote the enrichment of *Yersinia* in the gut microbiota through trophic transfer. Moreover, *Yersinia* is known to be more abundant in forest soils compared to open habitats such as grasslands, and the higher forest coverage in BX likely provides a substantial environmental reservoir for this genus [51]. Furthermore, the relatively lower temperatures in BX may further facilitate the persistence of *Yersinia*, as some species exhibit psychrotolerant characteristics and are capable of growth at temperatures as low as 0–5 °C [52].

In the FS population, *Sergentomyia* represents the most abundant dietary taxon and shows significant positive correlations with *Paracoccus* and *Acinetobacter* in the gut microbiota. Previous studies on *Sergentomyia babu* have revealed a highly diverse gut bacterial community comprising over 300 genera, with *Paracoccus* and *Acinetobacter* among the dominant taxa [53]. These genera are also commonly reported as core members of the microbiota in various insect species. The frequent consumption of *Sergentomyia* by frogs in the FS region may therefore facilitate the co-introduction of these bacteria into the host gut through trophic transfer, leading to their concurrent enrichment in the gut microbiota. This pattern highlights the potential role of prey-associated microbiota as an important source of gut microbial assembly in amphibian hosts.

### 4.3. Gut Microbiota as a Mediator of Host Phenotypic Plasticity

Across all populations, the gut microbiota of *R. dybowskii* was dominated by Proteobacteria, Actinobacteria, Firmicutes, and Bacteroidetes, a pattern consistent with previous studies in amphibians [43]. However, marked differences in their relative abundances among regions indicate functional divergence of microbial communities under distinct ecological conditions. Rather than reflecting taxonomic variation alone, these shifts likely correspond to differences in metabolic capacity, particularly in pathways related to protein digestion, carbohydrate utilization, and short-chain fatty acid (SCFA) production.

In the DD population, the enrichment of taxa such as *Arthrobacter* and *Robinsoniella* suggests a microbiota functionally adapted to processing chitin-rich and complex organic substrates. This enhanced capacity for nutrient extraction may contribute to improved energy assimilation efficiency, thereby supporting the greater body size and growth-related trait values observed in this population. The FS population exhibited higher microbial diversity and enrichment of taxa associated with versatile metabolic capabilities, including *Pseudomonas*, *Paracoccus*, and *Acinetobacter*. These bacteria are involved in the degradation of proteins and polysaccharides, as well as in nitrogen cycling, suggesting an adaptive response to a more heterogeneous and terrestrial-based diet. Such diversity does not necessarily translate into greater growth efficiency, as reflected by the relatively lower body size in this population. In the BX population, the presence of taxa such as *Yersinia* and *Eubacterium* indicates a microbiota shaped by both environmental filtering and dietary inputs. While some of these bacteria contribute to carbohydrate fermentation and maintenance of gut anaerobic conditions, others may reflect increased exposure to environmental microbes associated with forest habitats. This combination suggests a microbiota that is structurally distinct but functionally intermediate between the DD and FS populations. Specifically, microbial communities adapted to high-input, resource-pulse diets (as in DD) may enhance host growth through efficient substrate utilization, whereas communities associated with more diverse but lower-intensity resource environments (as in FS and BX) may prioritize metabolic flexibility over efficiency.

## 5. Conclusions

This study demonstrates that geographic variation among DD, FS, and BX populations of *Rana dybowskii* leads to pronounced differences in dietary composition and gut microbiota, which are closely associated with variation in host morphology. The Dandong population, characterized by a diet dominated by chironomid prey, exhibited a distinct microbial profile linked to efficient nutrient utilization and showed greater body size and growth-related trait values. In contrast, the more diverse but terrestrially oriented diets in Fushun and Benxi were associated with higher microbial diversity and the enrichment of environmentally derived and opportunistic taxa. Our findings are consistent with a framework in which environmental heterogeneity shapes dietary structure, diet covaries with gut microbial composition, and these factors together covary with host phenotypic differentiation. This study is consistent with the gut microbiome being one of several mediators linking ecological variation to host adaptive responses. We note, however, that this observational study is correlational: the proposed diet–microbiota–phenotype causal chain remains a hypothesis, and geographically varying factors such as individual age, hibernation duration, size at metamorphosis, and translocation history may independently influence both body size and diet. Direct experimental manipulations of diet and microbiota in controlled settings are needed to establish causality.

## Figures and Tables

**Table 1:** Geographic information, habitat characteristics, environmental parameters, and sample sizes of *Rana dybowskii* populations from three regions in Liaoning Province, China.

Region	Coordinates (°E, °N)	Habitat Type	Environmental Factors				Sample Number (Female: Male)		
			Average Rainfall (mm)	Average Temperature (°C)	Forest Coverage Rate (%)	Average Sunshine (hours)	Morphology Determination	16S rRNA Sequencing	DNA Metabarcoding
Benxi (BX)	124.90, 41.25	Semi-natural montane forest–wetland system	950.5	8.7	78.94	2055.2	362 (262:100)	6 (3:3)	10 (5:5)
Dandong (DD)	124.25, 40.74	Natural forest–wetland system with low industrial influence	1322.1	10.1	74.50	2188.1	478 (245:233)	6 (3:3)	10 (5:5)
Fushun (FS)	125.08, 42.33	Human-modified forest–agricultural wetland mosaic	786.7	7.2	70.30	2367.2	414 (211:203)	6 (3:3)	10 (5:5)

**Table 2:** Morphological traits of *Rana dybowskii* from three regions (BX, FS, DD), separated by sex.

Morphological Traits	BX		FS		DD	
	Female (*n* = 262)	Male (*n* = 100)	Female (*n* = 211)	Male (*n* = 203)	Female (*n* = 245)	Male (*n* = 233)
Body weight (g)	24.2 ± 5.69 ^c^	17.30 ± 5.98 ^c^	24.54 ± 5.83 ^c^	17.95 ± 4.32 ^bc^	26.29 ± 6.17 ^b^	18.40 ± 3.63 ^b^
Snout-vent length (mm)	62.73 ± 4.37 ^c^	57.56 ± 6.73 ^c^	62.89 ± 4.89 ^c^	57.25 ± 4.45 ^c^	64.68 ± 4.84 ^b^	58.93 ± 4.23 ^b^
Head length (mm)	19.39 ± 1.61 ^c^	17.95 ± 2.26 ^c^	19.37 ± 1.39 ^c^	18.45 ± 1.32 ^b^	19.86 ± 1.85 ^b^	18.73 ± 1.27 ^b^
Head width (mm)	18.17 ± 1.52 ^c^	16.83 ± 2.09 ^c^	18.12 ± 1.45 ^c^	17.04 ± 1.77 ^c^	18.39 ± 1.43 ^c^	17.49 ± 1.66 ^b^
Snout length (mm)	8.46 ± 0.76 ^c^	7.69 ± 0.92 ^c^	8.41 ± 0.74 ^c^	8.11 ± 0.79 ^b^	8.85 ± 2.44 ^b^	8.11 ± 0.76 ^b^
Forearm and hand (mm)	26.78 ± 2.26 ^c^	26.27 ± 3.68 ^c^	26.71 ± 2.18 ^c^	26.01 ± 4.49 ^c^	26.97 ± 3.22 ^b^	26.58 ± 3.53 ^b^
Forearm width (mm)	5.46 ± 0.67 ^c^	6.90 ± 1.24 ^b^	5.37 ± 0.61 ^c^	6.63 ± 0.86 ^c^	5.80 ± 0.69 ^b^	7.10 ± 0.87 ^b^
Hindlimb length (mm)	105.19 ± 7.70 ^b^	99.75 ± 12.76 ^c^	102.79 ± 8.72 ^a^	101.26 ± 8.57 ^bc^	105.16 ± 8.35 ^b^	102.28 ± 7.86 ^b^
Shank length (mm)	34.15 ± 2.89 ^b^	31.66 ± 4.43 ^c^	33.32 ± 2.91 ^a^	31.85 ± 2.98 ^c^	34.13 ± 4.14 ^b^	33.04 ± 3.02 ^b^
Foot length (mm)	34.15 ± 2.82 ^c^	33.44 ± 4.35 ^c^	33.67 ± 2.87 ^c^	33.12 ± 3.05 ^c^	34.75 ± 2.98 ^b^	33.89 ± 2.91 ^c^
Thigh length (mm)	31.58 ± 2.89 ^c^	29.29 ± 4.54 ^c^	31.37 ± 2.72 ^c^	30.16 ± 2.71 ^b^	32.35 ± 3.01 ^b^	30.67 ± 3.31 ^b^

Note: Values are presented as mean ± SD. Different lowercase letters within a column indicate significant differences among the three populations at *p* < 0.05 (Duncan’s multiple comparison test); values sharing a letter are not significantly different.

## Data Availability

The raw reads have been deposited in the NCBI database (BioProject number PRJNA1456344).

## Supplementary Materials

The following supporting information can be downloaded at: https://www.mdpi.com/article/10.3390/ani16213433/s1, Figure S1: Map of the three sampling sites of adult *R. dybowskii* in Liaoning Province, China; Figure S2: Principal Coordinates Analysis (PCoA) based on Bray–Curtis distances showing distinct clustering of gut microbial communities among the three geographic populations of *R. dybowskii*; Figure S3: Relative abundance of prey species in the diet of the three geographic populations of *R. dybowskii*; Figure S4: Correlation analysis between dominant dietary taxa and gut bacterial species in the three geographic populations of *R. dybowskii*; Table S1: Effects of Snout-vent length (SVL) and weight on gut microbial composition; Table S2: Effects of Snout-vent length (SVL) and weight on dietary arthropod composition.
