# Supplementary material for: Diet-Associated Gut Microbiome Divergence Contributes to Phenotypic Differentiation Among Geographically Structured Populations of Rana dybowskii
Source: Animals (Basel). 2026 Oct 30;16(21):3433. doi: 10.3390/ani16213433 (PMC13762279; doi:10.3390/ani16213433)
Supplement: Supplementary file 1 [file animals-16-03433-s001.zip › Figure S1.pdf]

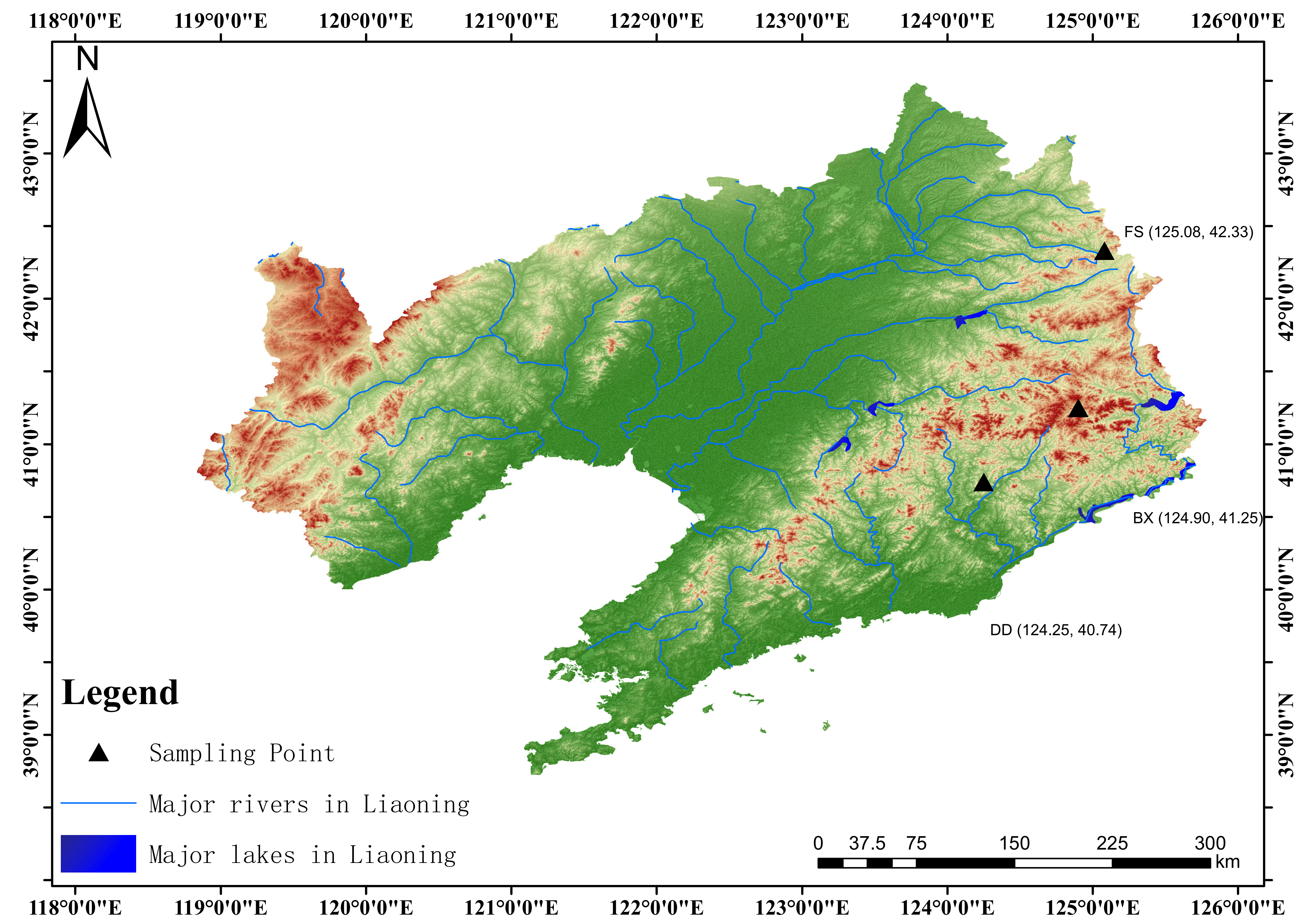

118°0'0"E
119°0'0"E
120°0'0"E
121°0'0"E
122°0'0"E
123°0'0"E
124°0'0"E
125°0'0"E
126°0'0"E
43°0'0"N
42°0'0"N
41°0'0"N
40°0'0"N
39°0'0"N
N
FS (125.08, 42.33)
BX (124.90, 41.25)
DD (124.25, 40.74)
Legend
Sampling Point
Major rivers in Liaoning
Major lakes in Liaoning
0 37.5 75 150 225 300
km
