# Supplementary material for: Diet-Associated Gut Microbiome Divergence Contributes to Phenotypic Differentiation Among Geographically Structured Populations of Rana dybowskii
Source: Animals (Basel). 2026 Oct 30;16(21):3433. doi: 10.3390/ani16213433 (PMC13762279; doi:10.3390/ani16213433)
Supplement: Supplementary file 1 [file animals-16-03433-s001.zip › Figure S2.pdf]

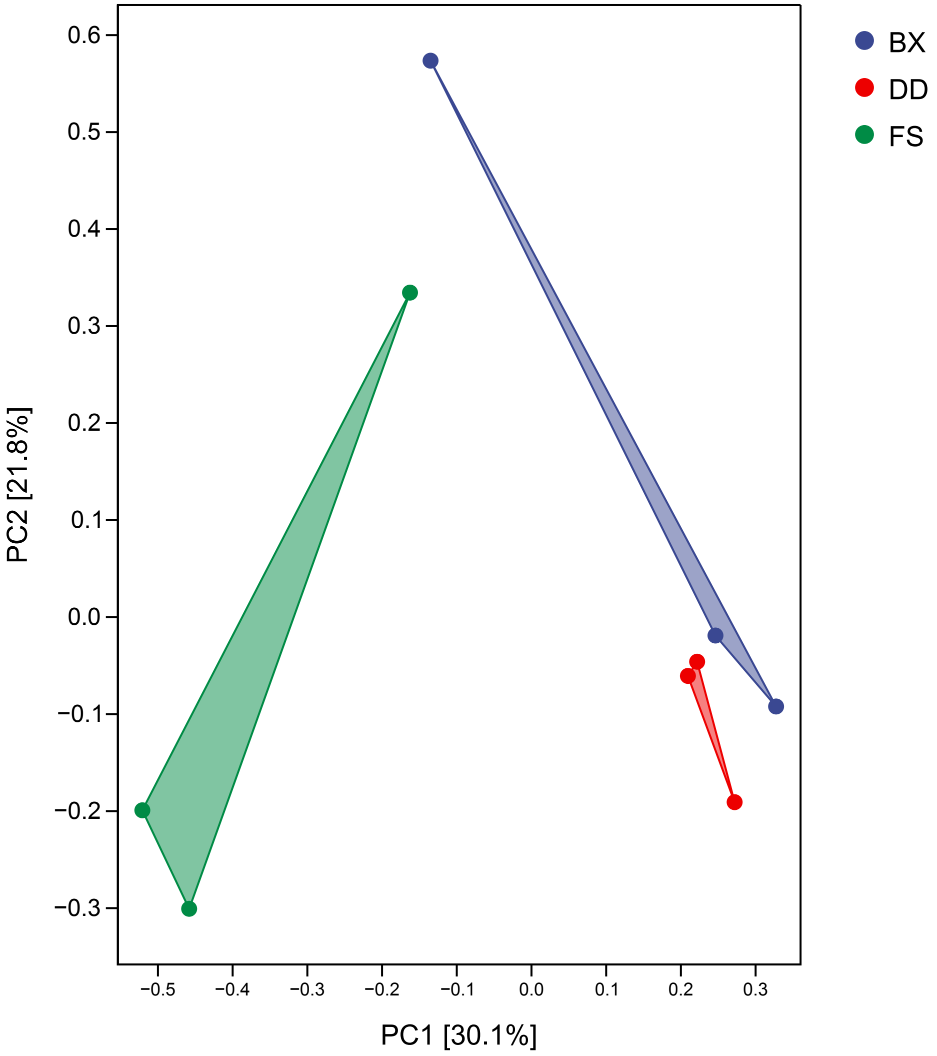

BX
DD
FS
PC2 [21.8%]
PC1 [30.1%]
0.6
0.5
0.4
0.3
0.2
0.1
0.0
−0.1
−0.2
−0.3
−0.5
−0.4
−0.3
−0.2
−0.1
0.0
0.1
0.2
0.3
