# Supplementary material for: Diet-Associated Gut Microbiome Divergence Contributes to Phenotypic Differentiation Among Geographically Structured Populations of Rana dybowskii
Source: Animals (Basel). 2026 Oct 30;16(21):3433. doi: 10.3390/ani16213433 (PMC13762279; doi:10.3390/ani16213433)
Supplement: Supplementary file 1 [file animals-16-03433-s001.zip › Figure S3.pdf]

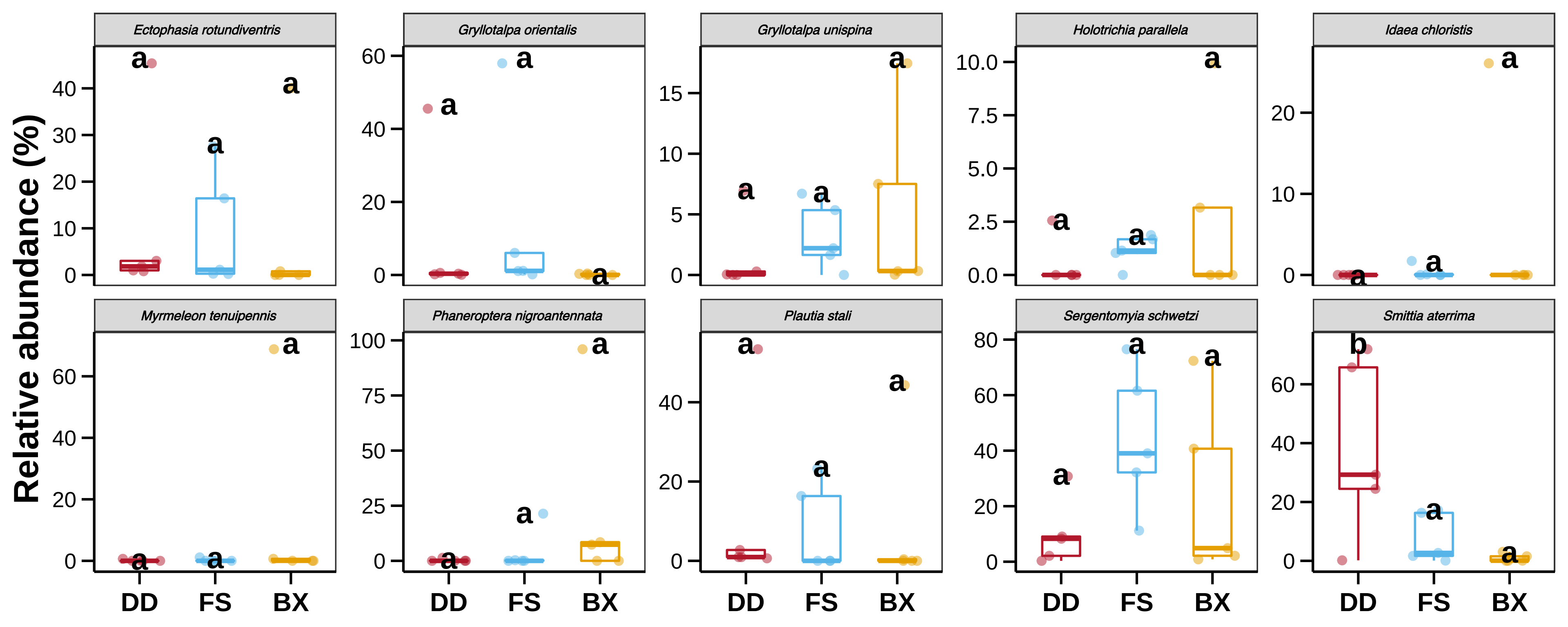

Relative abundance (%)
Ectophasia rotundiventris
Gryllotalpa orientalis
Gryllotalpa unispina
Holotrichia parallela
Idaea chloristis
Myrmeleon tenuipennis
Phaneroptera nigroantennata
Plautia stali
Sergentomyia schwetzi
Smittia aterrima
DD
FS
BX
