# Supplementary material for: Diet-Associated Gut Microbiome Divergence Contributes to Phenotypic Differentiation Among Geographically Structured Populations of Rana dybowskii
Source: Animals (Basel). 2026 Oct 30;16(21):3433. doi: 10.3390/ani16213433 (PMC13762279; doi:10.3390/ani16213433)
Supplement: Supplementary file 1 [file animals-16-03433-s001.zip › Figure S4.pdf]

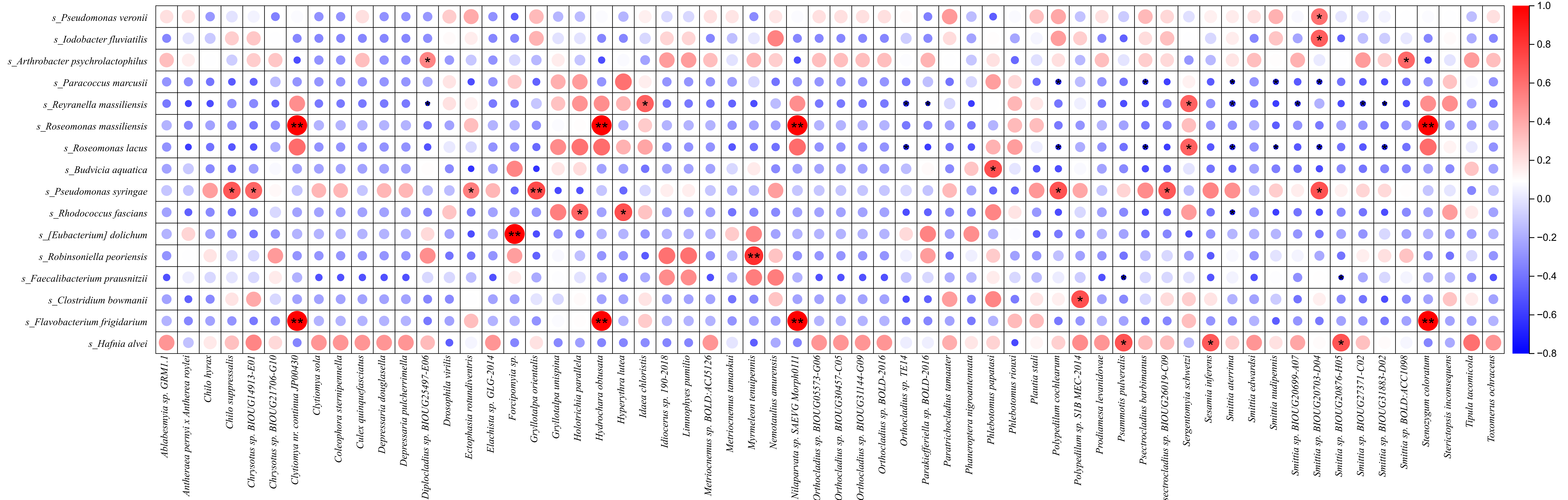
s_Pseudomonas veronii
s_Iodobacter fluviatilis
s_Arthrobacter psychrolactophilus
s_Paracoccus marcusii
s_Reyranella massiliensis
s_Roseomonas massiliensis
s_Roseomonas lacus
s_Budvicia aquatica
s_Pseudomonas syringae
s_Rhodococcus fascians
s_[Eubacterium] dolichum
s_Robinsoniella peoriensis
s_Faecalibacterium prausnitzii
s_Clostridium bowmanii
s_Flavobacterium frigidarium
s_Hafnia alvei
Ablabesmyia sp. GRM1.1
Antheraea pernyi x Antheraea roylei
Chilo hyrax
Chilo suppressalis
Chrysotus sp. BIOUG14913-E01
Chrysotus sp. BIOUG21706-G10
Clytiomya nr. continua JP00430
Clytiomya sola
Coleophora sternipennella
Culex quinquefasciatus
Depressaria douglasella
Depressaria pulcherrimella
Diplocladius sp. BIOUG25497-E06
Drosophila virilis
Ectophasia rotundiventris
Elachista sp. GLG-2014
Forcipomyia sp.
Gryllotalpa orientalis
Gryllotalpa unispina
Holotrichia parallela
Hydrochara obtusata
Hyperythra lutea
Idaea chloristis
Idiocerus sp. 190-2018
Limnophyes pumilio
Metriocnemus sp. BOLD:ACJ5126
Metriocnemus tamaokui
Myrmeleon tenuipennis
Nemotaulius amurensis
Nilaparvata sp. SAEVG Morph0111
Orthocladius sp. BIOUG05573-G06
Orthocladius sp. BIOUG30457-C05
Orthocladius sp. BIOUG31144-G09
Orthocladius sp. BOLD-2016
Orthocladius sp. TE14
Parakiefferiella sp. BOLD-2016
Paratrichocladius tamaater
Phaneroptera nigroantennata
Phlebotomus papatasi
Phlebotomus riouxi
Plautia stali
Polypedilum cochlearum
Polypedilum sp. S1B MEC-2014
Prodiamesa levanidovae
Psammotis pulveralis
Psectrocladius barbimanus
Psectrocladius sp. BIOUG26019-C09
Sergentomyia schwetzi
Sesamia inferens
Smittia aterrima
Smittia edwardsi
Smittia nudipennis
Smittia sp. BIOUG20699-A07
Smittia sp. BIOUG20703-D04
Smittia sp. BIOUG20876-H05
Smittia sp. BIOUG27371-C02
Smittia sp. BIOUG31883-D02
Smittia sp. BOLD:ACC1098
Stenozygum coloratum
Stictopsis inconsequens
Tipula tacomicola
Toxomerus ochraceus
1.0
0.8
0.6
0.4
0.2
0.0
−0.2
−0.4
−0.6
−0.8
